# Clinical outcomes and prognostic factors of stereotactic body radiation therapy for intrahepatic cholangiocarcinoma

**DOI:** 10.18632/oncotarget.19972

**Published:** 2017-08-07

**Authors:** Ze-Tian Shen, Han Zhou, Ao-Mei Li, Bing Li, Jun-Shu Shen, Xi-Xu Zhu

**Affiliations:** ^1^ Department of Radiation Oncology, Jinling Hospital, Medical School of Nanjing University, Nanjing, China

**Keywords:** intrahepatic cholangiocarcinoma, stereotactic body radiotherapy, CyberKnife, unresectable, Synchrony

## Abstract

Stereotactic body radiation therapy (SBRT) has been an emerging non-invasive treatment modality for patients with intrahepatic cholangiocarcinoma (ICC) when surgical treatment cannot be applied. The CyberKnife^®^ is a SBRT system that allows for real-time tracking of the tumor. The purpose of this study was to evaluate the clinical outcomes and prognostic factors for ICC patients receiving this treatment. Twenty-eight patients with ICC were enrolled in the present study. The median prescription dose was 45 Gy (range, 36-54 Gy), fractionated 3 to 5 times with a 70% to 92% isodose line. Local control, overall survival, progression-free survival and toxicity were studied. The median follow-up time was 16 months (3-42 months). Based on modified Response Evaluation and Criteria in Solid Tumors (mRECIST), response rate and disease control rate of SBRT in ICC were 46.4% (13/28) and 89.3% (25/28), respectively. Median overall survival was 15 months (95% CI, 7.22-22.78). 1- and 2-years survival rates were 57.1% and 32.1%, and 1- and 2- years Progression-free Survival rates were 50.0 % and 21.4 %. Multivariate analysis revealed that number of lesions (solitary vs. multiple nodules), CA19-9 levels (≤37 U/mL vs. 37-600/>600) and TNM stage (AJCC stage) were independent prognostic factors for ICC patients treated with SBRT. Toxicity was mostly transient and tolerable. No greater than grade 3 toxicity was observed. These results suggested that CyberKnife SBRT might be a good alternative treatment for unresectable ICC.

## INTRODUCTION

Intrahepatic cholangiocarcinoma (ICC) is the second most common primary hepatic malignancy after hepatocellular carcinoma (HCC) [1], accounting for 10%–15% of primary liver cancers. Since 1973, ICC has increased by 165% in 30 years in the USA [2]. China lacks reports in this regard, but the number of patients for the clinical diagnosis of ICC has been increasing every year.

For ICC, surgical resection has historically been considered the only curative option. However, only about one-third of patients present with resectable disease [3, 4]. Non-surgical options for patients with unresectable ICC include systemic chemotherapy [5], biliary drainage [6], external beam radiotherapy (EBRT) [7], transarterial chemoembolization (TACE) [8], and photodynamic therapy (PDT) [9–10]. However, ICC responds poorly to these therapies, so these palliative options are of limited benefit. Although EBRT is the most common local therapy utilized for patients without metastatic disease, the majority of patients eventually progress because of the low tolerance of the liver to radiation and the difficulty in localizing tumors as a result of organ motion [11].

As a new means of radiotherapy, CyberKnife avoids the errors caused by respiratory movement through synchronous respiratory tracking technology. By implanting gold fiducials in or around the tumors, tumor movement synchronized with respiratory motion can be truly tracked, more accurately giving the tumor higher doses, while reducing the dose to normal tissues [12–13]. With the advent of advanced imaging techniques and robotic image-guided radiation technologies, the CyberKnife SBRT achieves excellent conformality and significantly improves the therapeutic dose of localized liver lesions. Although SBRT has been used clinically for more than 10 years, especially in HCC [14–17], few studies were reported in ICC. Therefore, we retrospectively analyzed the efficacy, toxicity, and prognostic factors of SBRT for 28 cases of patients with primary ICC.

## RESULTS

### Patients’ characteristics

Patients’ demographics and baseline characteristics were displayed in Table 1. A total of 28 ICC patients (18 males and 10 females), all confirmed by pathologic diagnosis, were collected in this study. All 28 patients did not have distant metastases and ascites. Eight patients had previously received TACE treatment before CyberKnife SBRT while the remaining 20 patients did not receive any other treatments. Eight of the 28 patients had more than one liver lesion, in which 5 patients had two lesions and 3 patients had three lesions. Of the 28 ICC patients, 19 patients had concomitant liver diseases in this study, among which 13 patients had hepatitis B, 2 patients had hepatitis C, and 6 patients had hepatolithiasis. Among patients with hepatitis B or C, 8 patients had cirrhosis. In the present study, 20 patients had Child-Pugh A (A5:8, A6:12) classification and 8 patients had Child-Pugh B (B7:3, B8:2, B9:3) classification.

**Table 1:** Patient and treatment characteristics

Characteristics	No. (%) or median [range]
Gender	
Male	18(64.3)
Female	10(35.7)
Age(years)	
≤60	11(39.3)
>60	17(60.7)
ECOG performance status	
0	16(57.1)
1	10(35.7)
2	2(7.2)
Liver disease (etiology)	
Hepatitis B	13(46.4)
Hepatitis C	2(7.1)
Cirrhosis	8(28.6)
Hepatolithiasis	6(21.4)
Unknown	9(32.1)
Child-Pugh classification	
A5	8(28.6)
A6	12(42.9)
B7	3(10.7)
B8	2(7.1)
B9	3(10.7)
Diameter(cm)	
≤5	6(21.4)
5-10	15(53.6)
>10	7(25.0)
Number of lesions	
Solitary	20(71.4)
Multiple nodules	8(28.6)
Tumor location	
Peripheral ICC^*^	22(78.6)
Central ICC	6(21.4)
Reason for inoperability	
Medical comorbidity	7
Surgically unresectable	16
Advanced age	5
CA19-9(U/mL)	
≤37	6(21.4)
37-600	8(28.6)
>600	14(50.0)
TACE	
Yes	8(28.6)
No	20(71.4)
AJCC stage(7th)	
II	6(21.4)
III	19(67.9)
IVA	3(10.7)

^*^ICC: intrahepatic cholangiocarcinoma.

### Local control rate

Of the 28 patients with unresectable ICC who underwent CyberKnife SBRT, three patients (3/28, 10.7%) achieved a complete response (CR) and 10 patients (10/28, 35.7%) achieved a partial response (PR) at the first follow-up, resulting in an objective response rate (RR) of 46.4%. Stable disease (SD) was observed in 12 patients (42.9%), with an overall disease control rate (DCR) of 89.3%. Especially, the response rates and disease control rates in patients receiving a biological effective dose (BED) of ≥100 Gy was higher than those receiving BED<100 Gy (response rates: 52.4% vs. 0%, *P*=0.023; disease control rates: 100% vs. 57.1%, *P*=0.011). The response and disease control rates in patients with solitary lesions were higher than those in patients with multiple nodules lesions (60% vs. 12.5%, *P*=0.038; 100% vs. 62.5%, *P*=0.017) (Table 2).

**Table 2:** Local outcome of CyberKnife on 28 patients with locally advanced ICC [n (%)]

Subgroup		RR^*^		*p*	DCR^**^		*p*
		+	-		+	-	
	n	No. (%)			No. (%)		
Total	28	46.4 (13/28)			89.3 (25/28)		
Tumor types, n (%)							
Peripheral ICC	22	50 (11/22)	50 (11/22)	**0.655**	95.5 (21/22)	4.5 (1/22)	**0.107**
Central ICC	6	33.3 (2/6)	66.7 (4/6)		66.7 (4/6)	33.3 (2/6)	
Diameter (cm)							
≤5, n (%)	6	83.3 (5/6)	16.7 (1/6)	**0.069**	83.3 (5/6)	16.7 (1/6)	**0.530**
>5, n (%)	22	36.4 (8/22)	63.6 (14/22)		90.9 (20/22)	9.1 (2/22)	
Intrahepatic lesions, n (%)							
Solitary	20	60 (12/20)	40 (8/20)	**0.038**	100 (20/20)	0 (0/20)	**0.017**
Multiple nodules	8	12.5 (1/8)	87.5 (7/8)		62.5 (5/8)	37.5 (3/8)	
TACE, n (%)							
Yes	8	62.5 (5/8)	37.5 (3/8)	**0.410**	87.5 (7/8)	12.5 (1/8)	**0.652**
No	20	40 (8/20)	60 (12/20)		90 (18/20)	10 (2/20)	
BED							
≤100Gy	7	0 (0/7)	100 (7/7)	**0.023**	57.1 (4/7)	42.9 (3/7)	**0.011**
>100Gy	21	52.4 (11/21)	47.6 (10/21)		100 (21/21)	0 (0/21)	

^*^RR: Response rate = Complete response (CR) + Partial response (PR).

^**^DCR: Disease control rate = CR + PR + stable disease (SD).

### Long-term survival and prognostic factors

**Figure 1:**
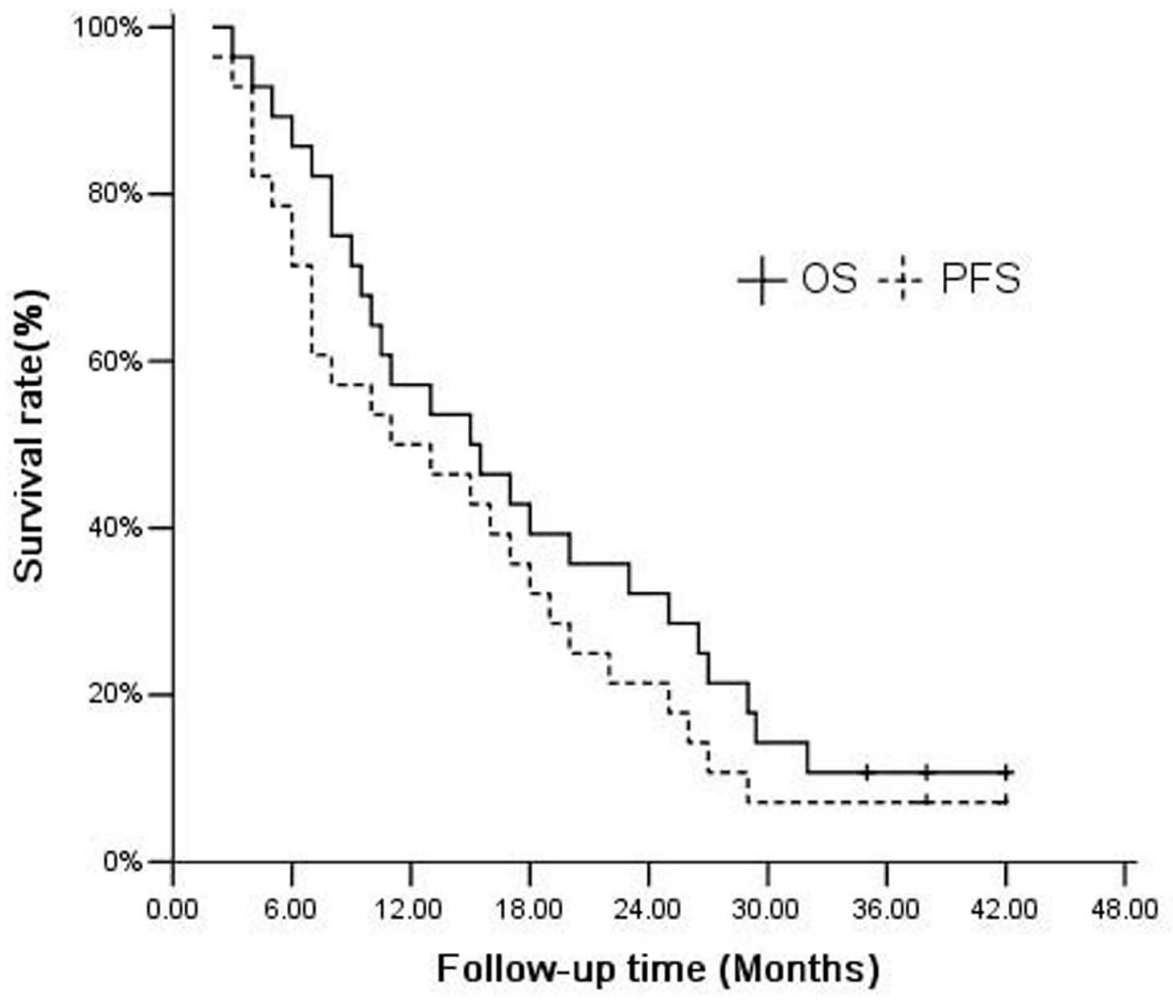
Kaplan–Meier curves for OS and PFS (n=28)

Among the 28 patients, the median follow-up time was 16 (3–42) months, and the median overall survival (OS) and progression-free survival (PFS) time were 15.0 months (95% CI, 7.22–22.78) and 11.0 months (95% CI, 1.93–20.08) respectively. Overall survival rate at 1- and 2-years was 57.1% and 32.1%, while 1- and 2- year Progression-free Survival (PFS) rates were 50.0 % and 21.4 % (Figure 1). The analysis of the prognostic factors was based on survival calculated from the start of CyberKnife SBRT. To further analyze the prognostic factors of ICC patients receiving CyberKnife SBRT, multivariate analysis was applied with 11 parameters, including Gender, Age, ECOG performance status, Child-Pugh classification, Diameter, Number of lesions, Tumor location, Reason for inoperability, CA19-9, AJCC stage and TACE. As indicated in Table 3 , number of lesions (HR, 5.444(95%CI, 1.446∼20.491), P=0.012), CA19-9 levels (HR, 0.018(95%CI, 0.001∼0.228), P=0.002) and TNM stage (HR, 2.096(95%CI, 1.111∼3.954), P=0.022) were independent prognostic factors for ICC patients receiving CyberKnife SBRT. Patients with solitary nodules, CA19-9≤37 U/mL or early clinical stage had better overall survival (*P* < 0.05).

**Table 3:** Prognostic factors for overall survival of 28 patients

Variables	*P*	HR	95.0%CI for HR	
			Lower	Upper
Gender	0.652	0.765	0.238	2.454
Age	0.381	2.139	0.390	11.729
ECOG performance status	0.150	0.453	0.154	1.331
Child-Pugh classification	0.964	1.042	0.170	6.390
Diameter	0.143	2.433	0.740	7.995
Number of lesions	0.012	5.444	1.446	20.491
Tumor location	0.495	1.059	0.898	1.250
Reason for inoperability	0.389	0.999	0.998	1.001
CA19-9	0.002	0.018	0.001	0.228
AJCC stage	0.022	2.096	1.111	3.954
TACE	0.064	9.686	0.872	107.536

### Side effects

The Common Toxicity Criteria Adverse Events (CTCAE 4.0) radiation injury-grading criterion was used to evaluate radiation injury in the present study. The main side effects of treatment include mild fatigue, anorexia, nausea, vomiting, mildly elevated liver enzymes, and bone marrow suppression (Table 4). Grade 1-2 anorexia was the most common toxicity and was developed in 42.8 % of the patients. Of the 28 patients, the grade 3 toxicities included grade 3 gastrointestinal ulcers (antral ulcer confirmed by endoscopy), liver enzyme toxicity and bone marrow suppression. Toxicities greater than grade 3 were not observed. Six patients with central region lesions experienced serious gastrointestinal reaction, in which one patient experienced grade 3 vomiting and two experienced grade 2 nausea and anorexia respectively.

**Table 4:** Side effects of 28 patents with ICC in the treatment of CyberKnife SBRT [n(%)]

CTC 4.0 toxicity^*^	1	2	3	4	5
	n (%)	n (%)	n (%)	n (%)	n (%)
Fatigue	4 (14.3%)	3 (10.7%)	0 (0)	0 (0)	0 (0)
Lethargy	5 (17.9%)	2 (7.1%)	0 (0)	0 (0)	0 (0)
Pleural effusion	2 (7.1%)	1 (3.6%)	0 (0)	0 (0)	0 (0)
Gastrointestinal					
Nausea	8 (28.6%)	3 (10.7%)	1 (3.6%)	0 (0)	0 (0)
Anorexia	10 (35.7%)	2 (7.1%)	2 (7.1%)	0 (0)	0 (0)
Vomiting	5 (17.9%)	2 (7.1%)	1 (3.6%)	0 (0)	0 (0)
Duodenal ulcer	2 (7.1%)	0 (0)	0 (0)	0 (0)	0 (0)
Gastric ulcer	2 (7.1%)	1 (3.6%)	1 (3.6%)	0 (0)	0 (0)
Hepatic					
ALT	3 (10.7%)	4 (14.3%)	2 (7.1%)	0 (0)	0 (0)
AST	5 (17.9%)	6 (21.4%)	1 (3.6%)	0 (0)	0 (0)
Albumin	1 (3.6%)	6 (21.4%)	1 (3.6%)	0 (0)	0 (0)
ALP	2 (7.1%)	3 (10.7%)	1 (3.6%)	0 (0)	0 (0)
Bilirubin	2 (7.1%)	4 (14.3%)	2 (7.1%)	0 (0)	0 (0)
Bone marrow					
WBC	7 (25%)	3 (10.7%)	1 (3.6%)	0 (0)	0 (0)
Hb	3 (10.7%)	2 (7.1%)	1 (3.6%)	0 (0)	0 (0)
PLT	10 (35.7%)	2 (7.1%)	1 (3.6%)	0 (0)	0 (0)

^*^CTC 4.0: The Common Toxicity Criteria Adverse Events (version 4.0).

When the tumor size smaller than 5 cm, toxicities greater than grade 1 were not observed in which two cases developed grade 1 fatigue and nausea. Toxicities greater than grade 1 were all observed in the tumor size larger than 5cm. Especially, when the tumor size larger than 10 cm, it was easier to experience fatigue, anorexia and nausea. Classic Radiation induced liver disease (RILD) was not observed in the whole group of patients. Two patients developed non-classic RILD. After symptomatic treatment, the cases recovered smoothly. There was no treatment-related death.

## DISCUSSION

Surgical resection has been considered a preferred treatment modality for long-term control of some early stage ICCs. However, most patients have lost the opportunity of operation at diagnosis. Although TACE [8] and radiofrequency ablation (RFA) [18] have been widely used in ICC, the special features of ICC (fibrosis, lack of blood supply, and large tumor volume) limit their application. Currently, systematic chemotherapy with or without local treatment [5] is a common strategy for inoperable ICC, and radiation therapy [7] is one of the most important local treatments.

In recent years, several studies have been reported on conventional radiotherapy used to treat ICC. Given the small sample size and most studies involved other types of cholangiocarcinoma, the clinical benefits of radiotherapy in ICC remains uncertain. In general, the response rate of radiotherapy on biliary tract cancer is 40%–45% [7, 19, 20], which was due to the technical limitations. The efficacy of conventional radiotherapy, such as three-dimensional conformal radiotherapy and intensity-modulated radiation therapy, remains to be improved.

The appearance of SBRT therapeutic modalities compensates for the deficiencies of conventional radiotherapy. Nowadays, SBRT is increasingly applied to inoperable liver cancer. However, reports of SBRT for treating liver tumors were mainly about primary hepatocellular carcinoma and liver metastases [15, 21, 22], few reports were about ICC. Most of the studies were retrospective with small cohorts mixing ICC and HCC types simultaneously [23, 24], so the results of the studies could not completely reflect the therapeutic effect of ICC. Barney [25] reported the results of 10 patients with only advanced cholangiocarcinoma treated with SBRT; a total of 12 lesions, 6 cases of primary, and six cases of recurrence were reported, with a median dose of 55 Gy (45–60 Gy), median follow-up time of 14 months (2–26 months), local control rate of 100%, six-month survival rate of 83%, and 12-month survival rate of 73%. In this study, SBRT achieved good clinical effects for ICC, but the severe side effects (such as Grade 5 liver failure) limited its further clinical application.

The most comparable series to the present study is from Mahadevan et al [26]. They reported on 34 patients with 42 lesions containing 31 intrahepatic and 11 hilar lesions. The median SBRT dose was 30Gy in three fractions and the median follow-up was 38 months (range 8-71months). Their actuarial 1-year local control rate was 88%. The median OS and PFS were 17 months and ten months. While in the present study, 1-year local control rate (89.3%), median OS (15 months) and median PFS (11 months) were comparable with Mahadevan’s study.

The local control rate was significantly higher than the previous reports, suggesting that the local control rate of SBRT for treating ICC was at a higher level, even for patients who had previously received TACE treatment. In this study, lesions greater than 5 and 10 cm accounted for 78.6% and 25%, respectively, and the center of large tumor necrosis during follow up reduced efficiency. Affected by the age of patients, physical condition, extent, location of the lesion, and other factors, we only administered the palliative treatment dose. Our study found a higher rate of local control and efficiency in the BED ≥ 100 Gy group, suggesting that BED may be an important factor of SBRT, which was similar to previous reports [27]. Notably, there were no uniform standards for the total dose, treatment times, single dose, and interval of CyberKnife SBRT to treat inoperable ICC. All these factors require further study.

For the 28 patients enrolled in this study, the median survival time was 15.0 months. The one-year OS and PFS rate were 57.1% and 50.0%, and the two-year OS and PFS rate were 32.1% and 21.4%, respectively. These values were better than the data reported [23–26]. Multivariate analysis showed that the main factors affecting the long-term survival of ICC patients receiving SBRT were the number of lesions (solitary vs. multiple nodules), CA19-9 levels (≤37 U/mL vs. 37-600/>600) and TNM stage (AJCC stage). For patients with multiple nodules and late clinical stages, many surrounding normal tissues restrict the distribution of the dose line, resulting in the uneven distribution of the dose and dose cold spots. Meanwhile, the tumor’s internal blood supply is worse based on ICC. High levels of necrosis and tumor tissue hypoxia lead to radiation tolerance, and high-dose radiation therapy is still very difficult to overcome. Moreover, more advanced stage of the disease often means being prone to distant metastasis, thereby making the prognosis worse.

Given that liver tumors move with breathing, CyberKnife SBRT avoids the errors caused by respiratory movement through synchronous respiratory tracking technology. Considering the high single dose, we strictly limited the irradiated dose (stomach, duodenum, and kidney) of surrounding normal tissues. The main side effects of SBRT were mild fatigue, anorexia, nausea, vomiting, mild elevated liver enzymes, and bone marrow suppression, which could be improved and returned to normal after positive symptomatic treatment. After more than two years of observation, no greater than grade 3 long-term radiation-related toxicities was occurred, suggesting that the treatment was relatively safe.

In the latest NCCN guideline, systematic chemotherapy was also one option for the treatment of unresectable ICC. Median survival time for patients who undergo at least 4 cycles of chemotherapy (Gemcitabine-based combination regimens or 5-FU–based regimens) ranges from 6 to 14 months, with PFS time ranges from 2.3 to 8 months [5, 28]. However, in the present study, the median OS and PFS time of ICC patients treated by CyberKnife SBRT were 15.0 months and 11.0 months, which were significantly higher than that reported in the chemotherapy series.

In conclusion, our findings demonstrated that SBRT for inoperable ICC could achieve a high local control rate and one- and two-year survival rates. The toxicity could be tolerated and adverse reactions greater than grade 3 were not seen. Future studies should further expand the sample size. Optimal radiation dose and fractionation for future SBRT treatment is also needed to be studied.

## MATERIALS AND METHODS

### Ethics statement

Patients treated with CyberKnife SBRT were carried out in strict accordance with the procedure approved by Ethic Committee of Jinling Hospital. Patients have provided their written informed consents to receive the CyberKnife SBRT and agreed with their records to be used in this study.

### General information

From March 2009 to September 2012, 28 patients participated in a retrospective study at the CyberKnife center, Jinling Hospital. Patients were included based on the following criteria: (1) histologically proven diagnosis of ICC; (2) Patients were eligible for treatment if they had inoperable ICC according to more than two liver surgery experts; (3) Eastern Cooperative Oncology Group(ECOG) performance status of≤2; (3)Child-Pugh score≤7; (4) adequate hematology, including an absolute neutrophil count >1.5×10^9^/L, a platelet count >50×10^9^/L, renal function with creatinine level <2.0 mg/dl; (5) patients with no previous experience of radiotherapy; (6)patients not showing extrahepatic metastases; (7)the normal liver volume of more than 700 cc. All patients were treated with SBRT in Department of Radiation Oncology of Jinling Hospital and had signed a written informed consent for treatment. This study was approved by the institutional review board.

### Fiducial marker placement

All 28 patients were treated using a CyberKnife SBRT system (Accuray, USA). All patients were treated via respiration synchronous tracking (Synchrony); and three to six markers (size of 6.0 mm ×0.8 mm) were implanted within or around the tumor using a CT-guided 19G needle. The minimum distance should be >2 cm, and the angle should be >15° between two markers. CT scan was performed to observe whether the markers were in their proper positions or to detect the presence of pneumothorax 2 hours after implantation. A CT scan was performed again at 7–10 days after implantation. In the process of liver puncture, three cases developed grade 2 complications with less than 30% pneumothorax who recovered within 2 days via suction through a fine-needle puncture. Eight cases developed grade 1complications, consisting mostly of transient increases in blood pressure, local pain and hemorrhage. No tumor seeding was detected while grade 1 hemorrhage through needle passage was common.

### Positioning and target delineation

Patients were in the supine position with the body fixed with a vacuum pad. Spiral CT (Brilliace Big Bore 16 CT Philips Germany) scanning was conducted with a slice thickness of 1 mm. Hepatic scanning consisted of three phases (arterial phase, venous phase, and equilibrium phase). Hepatic scans covered 15 cm above and below the lesions. In order to better delineate tumor volumes, a set of MRI of liver was arranged in all patients, hepatic or delayed phases of MRI were fused with the planning CT scan for contouring, other phase images of MRI were used as reference. The gross target volume (GTV) and PTV were determined according to the tumor volume. We added a 5 mm margin to the GTV to account for residual inaccuracy of Synchrony. The prescription dose was defined as 100% of the GTV dose. The total PTV dose was not less than 95% of the prescription dose.

In cases of multiple tumors, we have used one center for each tumor. The center located in the geometric center of the markers. But, the lesions were included based on the following criteria: 1.At least one marker was implanted within each lesion; 2.The distance between each lesion was not larger than 3cm; 3. 4D-CT was used to measure the position and motion of the lesions. One additional margin (1-2mm) was planned on the basis of the original PTV of one lesion for residual inaccuracy of Synchrony. 4. For multiple lesions, the total PTV dose was not less than 95% of the prescription dose. 5. The irradiation dose of the normal liver should be in accordance with the criteria of dose constraints.

### Treatment mode and methods

Before treatment, a respiratory monitoring device was used to detect the position of the infrared generator placed on the chest of the patient to create a dynamic respiratory rhythm. The X-ray kV digital images were obtained at different time points during the respiratory cycle to obtain the dynamics model between the gold seed fiducial (tumor) position and respiratory rhythm. The respiratory model was used to guide the accelerator to track the lesions within the liver and administer the dynamic radiation. The prescription dose of lesions was 36–54 Gy (median dose, 45 Gy) in three to five divided doses. The SBRT doses was converted into normalized total dose (NTD) at a fraction size of 2 Gy (NTD2Gy) using the linear quadratic equation (BED = total dose × (1 + dose per fraction/α/β), α/β =10 for early responding tissue, α/β = 3 for late responding tissue). The BED10 for SBRT ranges from 72 Gy to 124.8Gy (median 85.5Gy). The dosimetry indexes of the 28 ICC patients are summarized in Table 5.

**Table 5:** Dosimetry index of the 28 patients during CyberKnife SBRT

Item	Median (range)		
	Total	≤5cm	>5cm
Gross tumor volume (cc)	267.4(43.4-1302.8)	74.3 (43.4-123.4)	327.2 (232.7-1302.8)
Prescription dose (Gy)	45 (36-54)	48 (45-54)	42 (36-50)
Dose per fraction (Gy)	15(10-18)	16 (15-18)	13 (10-16)
Conformity index (CI^*^)	1.14 (1.02–1.32)	1.05 (1.02-1.10)	1.21 (1.13-1.32)
New conformity index (nCI^**^)	1.23 (1.12–1.45)	1.16 (1.12-1.24)	1.27 (1.24-1.45)
Coverage^***^(%)	92(85-100)	96 (92-100)	89 (85-95)
Number of beams (median)	136 (45–284)	78 (45-142)	147 (129-284)
Prescription isodose line (%)	78(72-90)	85 (82-90)	76 (72-84)

^*^CI (Conformity Index): The ratio of the tissue volume that received the prescription isodose or more to the tumor volume receiving the prescription isodose or more.

^**^nCI (New Conformity Index): The data of the CI multiplied by the ratio of the total tumor volume to the tumor volume receiving the prescription isodose or more.

^***^Coverage: The volume of the tumor that received greater than or equal to the prescribed dose divided by the total volume of the tumor times 100.

When the CyberKnife SBRT plan was designed, the single point maximum dose was used as the limit standard for serial organs, and the single maximum dose of part volume was used as the limit standard for parallel organs. Five fractions were used as the limit standard for single point dose. Since most of patients had 5 fractions, the limit standard for single dose was appropriately increased for those patients with less 5 fractions. The dose constraints of the normal liver (total liver minus cumulative GTV) were specified that a minimum volume of 700 ml should receive a total dose less than 15 Gy in 3-5 fractions. The other normal tissues actual received doses and limit standards are shown in Table 6.

**Table 6:** The standard of dose limitation in critical structures and its actual exposure dose

Critical structures	Total max dose (Gy)	D33.3^*^ (Gy)	Max dose(Gy) per fraction	Dose constraints^**^	
	Mean(range)	Mean(range)	Mean(range)	Volume	Dose
Stomach	10.2(5.4∼21.5)	—	2.4(1.8∼4.6)	Any point	6 Gy per fraction
Duodenum	11.8(4.8∼27.6)	—	2.8(1.6∼5.5)	Any point	6 Gy per fraction
Spinal cord	4.8(3.6∼11.2)	—	2.1(1.0∼3.6)	Any point	5Gy per fraction
Left kidney	—	4.2(1.6∼9.4)	1.6(1.2∼2.6)	<1/3 of the total volume	4 Gy per fraction
Right kidney	—	5.7(3.6∼17.2)	2.6(1.5∼3.7)	<1/3 of the total volume	4 Gy per fraction

*D33.3=33.3 percentage of Volume receiving the exposure dose.

**Dose constraints: Dose constraints for critical structures were taken 5 fractions as a standard.

### Follow up and evaluation

Abdominal enhanced CT scan or MRI was performed one month after SBRT completion. Patients were monitored every three months thereafter. Clinical monitoring was performed every day. The mRECIST [29] Criteria in Solid Tumors was used to evaluate treatment efficacy. Response rate (RR) = Complete response (CR) + Partial response (PR), whereas disease control rate (DCR) = CR + PR + Stable disease (SD). Overall survival (OS) and progression-free survival (PFS) were measured from the start of CyberKnife SBRT. Follow up was performed every three months for a total of three to 42 months, with a median follow up of 16 months. The final follow-up time was in September 2012.

The Common Toxicity Criteria Adverse Events (version 4.0) radiation injury-grading criterion was used to evaluate radiation injury. Radiation induced liver disease(RILD) was characterized by either (1) anicteric elevation in alkaline phosphatase(ALP) to greater than twice the upper normal level and nonmalignant ascites (classic RILD) or (2) elevation of transaminases to at least five times the upper limit of normal or pretreatment levels (non-classic RILD) within 4 months after completion of radiotherapy.

### Statistical analysis

SPSS 15.0 statistical software was applied for data analysis. The Kaplan–Meier method was used to analyze PFS and OS. The log-rank method was used to test the significance compared with the survival curves. Multivariate analysis of survival was carried out with Cox’s regression model. P values less than 0.05 were considered statistically significant.
